# Acidification of intracellular pH in MM tumor cells overcomes resistance to hypoxia-mediated apoptosis *in vitro* and *in vivo*

**DOI:** 10.3389/fonc.2023.1268421

**Published:** 2023-11-03

**Authors:** Gilberto Gastelum, Jeffry Kraut, Mysore Veena, Alisher Baibussinov, Christopher Lamb, Kylee Lyons, Eric Y. Chang, Patrick Frost

**Affiliations:** ^1^ Department of Research, Greater Los Angeles Veterans Administration Healthcare System, Los Angeles, CA, United States; ^2^ Breast Cancer Program, Lombardi Comprehensive Cancer Center, Department of Oncology, Georgetown University, Washington, DC, United States; ^3^ Department of Hematology/Oncology, University of California, Los Angeles, CA, United States; ^4^ Department of Research, San Diego Veterans Administration Healthcare System, San Diego, CA, United States

**Keywords:** bone marrow, tumor microenvironment, hypoxia, pH, apoptosis

## Abstract

**Introduction:**

Multiple myeloma (MM) is an incurable cancer of malignant plasma cells that engraft in the bone marrow (BM). It is more than likely that the poorly investigated physical parameters of hypoxia and pH in the tumor microenvironment (TME) is critical for MM survival. Here, we explore the effects of a hypoxic environment on pH regulation and its role in MM survival.

**Methods:**

We used in vitro models of MM, in which the culturing medium was modified to specific pH and pO2 levels and then measured the effects on cell survival that was correlated with changes in intracellular (pHi) and extracellular pH (pHe). In a MM xenograft model, we used PET/CT to study hypoxia-mediated effects on tumor growth.

**Results:**

Hypoxia-mediated apoptosis of MM cells is correlated with acidic intracellular pHi (less than < 6.6) that is dependent on HIF activity. Using a polyamide HIF responsive element binding compound, a carbonic anhydrase inhibitor (acetazolamide), and an NHE-1 inhibitor (amiloride) acidified the pHi and lead to cell death. In contrast, treatment of cells with an alkalization agent, Na-lactate, rescued these cells by increasing the pHi (pH > 6.6). Finally, treatment of mice with acetazolamide decreased cell growth in the tumor nodules.

**Discussion:**

Targeting hypoxia and HIF have been proposed as an anti-tumor therapy but the clinical efficacy of such strategies are modest. We propose that targeting the pHi may be more effective at treating cancers within a hypoxic TME.

## Introduction

Multiple myeloma (MM) is an incurable disease of malignant plasma cells that engrafts in the bone marrow (BM), makes up about 10-15% of all hematological malignancies, kills 12,500 Americans/year and is the most frequent hematological cancer to involve the skeleton (1). Pharmacological treatment of MM includes proteasome inhibitors, immunomodulatory (IMiDs) drugs, monoclonal antibodies and steroids that have greatly improved outcomes, but patients eventually relapse and ultimately die (2). The reasons why MM is so difficult to cure is unknown, but it may be related to the effects of pro-survival factors within the BM niche that support MM survival and progression. In solid tumors, there is an increasing focus on the role of low oxygen levels (hypoxia as defined when the O2 levels in a tissue fall below the level required for cellular survival) and subsequent pH regulation in the tumor microenvironment but the role of these factors in hematological malignances, like MM, are poorly understood. In this study, we examine how regulation of the intracellular and extracellular pH compartments is crucial for MM tumor survival under hypoxic conditions and further show that this is dependent on hypoxia inducible transcription factors (HIFs).

The BM is a complex tissue of cellular and noncellular components that regulate MM biology. Equally important, yet relatively poorly understood, are the physical characteristics of the BM, like oxygen (pO_2_) and pH levels. Whereas it is known that normal BM is hypoxic (pO_2_<32 mmHg) (3), engrafted MM tumors have even lower pO_2_ levels (4). In fact, low O_2_ conditions predispose tumor cells to utilize anaerobic glycolysis which results in increased lactic acid production and the concomitant generation of a high extracellular acid load (via the Warburg Effect). This generates a reversed pH gradient when compared to the more basic/neutral intracellular pH (5, 6). Paradoxically, hypoxia and the resultant acidic TME should be inimical to the survival of MM tumors, but this can also activate survival pathways that protect these cells from hypoxia-mediated apoptosis (7), promote chemoresistance (8), and facilitate tumor metastasis (9). We have previously shown that HIF activity is a critical “master-regulator” of the cellular response to hypoxia in the MM/BM environment, and that inhibiting the ability of HIF to induce the protective adaptive hypoxic responses can overcome resistance to hypoxia-mediated killing (10–13).

The regulation of the HIFα-subunits is dependent on the prolyl hydroxylase domain proteins (PHD) that target the HIFα-subunits for ubiquitination and degradation [For review see (14)]. The failure to maintain HIF regulation results in constitutive HIF activity that can result in heightened expression in the downstream target of HIFs. In several cancers, including MM, the PHD3 isoform is downregulated, resulting in higher levels of HIF2α and greater resistance to hypoxia-mediated cell death (13). Targeting HIF activity has shown some preclinical efficacy, but has not been especially effective in the clinic, likely due to redundant and complex regulatory pathways in the tumor cells. In this study we show that the combination of downregulated or inactivated PHD3 and increased activity of HIF supports the expression of several membrane-bound proteins known to influence pH regulation such as carbonic anhydrase IX, the Na^+^/H^+^ exchanger 1 (NHE1) and the monocarboxylate transporters (MCTs). These factors play a critical role in regulating MM sensitivity to hypoxia-mediated pH homeostasis that favors MM survival and proliferation.

## Materials and methods

### Cell lines and reagents

OPM-2, 8226, H929 and U266 human MM cell lines were purchased from ATCC and were validated using the Johns Hopkins Genetic Core Research Facility (Baltimore, MD) and cultured in a standard humidified incubator at 37°C and 5% CO_2_ (at atmospheric or “normoxic” pO_2_ ~ 22%). For hypoxic culturing conditions (at “hypoxic” conditions of 0.2%), MM cells were maintained in a humidified, 37°C and 5% CO_2_ environment using a dedicated HypOxygen (Grandpair, Heidelberg, Germany) hypoxia cell culture incubation chamber. The O_2_ levels were regularly tested and calibrated using the manufacturer’s protocol. HypOxygen workstation is a glove box design with an integral airlock system that allows for maintaining a set pO_2_ level while manipulating the cell cultures without removing them from the hypoxic environment. The cells lines were maintained in sterile RPMI-1640 medium (Gibco, Waltham, MA) supplemented with 10% FBS (Gibco), L-glutamine, nonessential amino acids (Gibco), Na-pyruvate (Gibco), penicillin-streptomycin (Gibco), and Fungizone (Gibco) unless noted otherwise. Testing for mycoplasma was performed using a mycoplasma PCR detection kit (Sigma-Aldrich, St Louis, MO). Acetazolamide, amiloride hydrochloride hydrate, and sodium L-lactate were purchased from Millipore-Sigma (Burlington, MA).

Amiloride and acetazolamide were initially dissolved into DMSO to make a working stock solution (acetalozamide was prepared at 1.0 M and amiloride at 0.1M). Working solutions were then prepared in media to final concentrations used. Sequence-specific DNA-binding pyrrole-imidazole polyamides were synthesized by solid-phase methods on Kaiser oxime resin (Nova Biochem, Billerica, MA) (15). The tested polyamide, HIF-PA, targets the sequence 5′-WTWCGW-3′ (W=A or T) and modulates a subset of hypoxia- induced genes, whilst the control polyamide (CO-PA) recognizes the non-HRE sequence 5′-WGGWCW-3′. HIF-PA and control polyamides were a kind gift from Dr. Peter Dervan as previous described (12). The hypoxiprobe-1 kit was purchased from HPI Inc (Burlington, MA, USA). Staurosporin (1 uM) (Sigma) was used as a positive control for apoptosis.

### Modified pH RPMI-8226 medium

In some experiments a modified RPMI-8226 media (based on a protocol described by Michl (16) was used in which we reduced the amount of added NaHCO_3_ buffer by half and titrated the medium to a pH of 6.7 (acidic) or 7.4 (control basic/neutral). Briefly, RPMI-1640 powdered medium (without HEPES or NaHCO_3_) was prepared by adding 5mM NaHCO_3_ and then titrated to a pH of 6.7 for the “acidic” medium or a pH 7.4 for the “neutral/basic” medium using 1N NaOH or HCl. After sterile filtering, this medium was supplemented with 10% FBS, L-glutamine, non-essential amino acids, Na-pyruvate, penicillin-streptomycin, and Fungizone (as described above) and the final pH of the medium was retested and adjusted using sterile NaOH and HCl as needed. The long-term stability of the pH was determined by culturing cell free medium at various pO_2_ levels (22%, 2% and 0.2%) in a humidified, 37°C and 5% CO_2_ standard or hypoxic incubator (as described above). Aliquots of the medium were tested at various time periods (up to 96 hours) and the changes in the pH were measured using a pH electrode (in triplicate).

### Transduction of 8226 cells

8226 MM cell lines were stably transduced with a wild-type PHD3 allele (8226PHD3) or empty vector control (8226EV) as previously described (13). Briefly, the PHD3 (also known egl-9 family hypoxia inducible factor 3 (EGLN3) open reading frame (ORF)-DKK (tag) (ref sequence NM_022073.3) was cloned into a lentivirus vector (OriGene, Rockville MD). Isogenic 8226 cells were infected with the viral particles (empty vector controls or PHD3) at a multiplicity of infection (MOI) of 10 and gentamycin selection was started 72 hours later. Successful transduction was confirmed by immunoblot for PHD3 or DKK expression. Small inhibitory RNA (siRNA) for HIF1α (Silencer Select siRNA ID# s6539, gene ID# 3091), HIF2α (Silencer Select siRNA ID# s6539, gene ID# 2034), and scrambled control RNA (Silencer Select negative control #1 siRNA), were purchased from Ambion (Grand Island, NY). Cells were transfected with siRNA using Lipofectamine-2000 (Life Technologies, Grand Island, NY).

### Measurement of extracellular and intracellular pH

Cells were seeded in 6 well plates (0.5X10^5^ cell/well) in either acidic (pH 6.7) or neutral/basic (pH7.4) modified medium, then cultured in either standard incubator (normoxic) or hypoxia chamber (hypoxic) conditions (as described above). Under all experimental conditions, the pO_2_ of the test medium was allowed to equilibrate in the incubation chamber (under normoxic or hypoxic conditions) for at least 24 hours prior to use. At various time points (up to 72 hours) aliquots of the medium were collected and the pHe measured using a pH electrode. The effects of pO_2_ on the pHi were measured using a Fluorometric pHi Assay Kit (Sigma-Aldrich) following the manufacturer’s protocol. In brief, 1X10^4^ cells were seeded in a 96-well flat bottom plate (Corning Costar), with clear bottoms and black sides, in modified RPMI-1640 complete medium (titrated to either 6.7 or 7.4 pH). The cells were incubated at the indicated pO_2_ (22% or 0.2% as described above) for 48 hours. Subsequently, the fluorescent pH indicator BCFL-AM was added and fluorescence (λ_ex_ = 490 nm; λ_em_ = 535 nm) was measured at 5 hours using a SynergyHT plate reader (BioTek, Winooski, VT).

### Apoptosis assays

Cellular apoptosis was measured by flow cytometry using a cleaved caspase-3 kit or an annexin V/PI kit (BD Biosciences, San Jose, CA). Approximately 1X10^6^ cells were cultured under various pH and pO_2_ conditions as described above. The cells were prepared following the manufacturer’s instructions and samples run on a FACS-Canto II (Becton-Dickenson) flow cytometer. The computer program FLOWJO was used to analyze flow cytometry data. In some experiments, we also performed immunoblots using an apoptosis antibody sampler (Cell Signaling Technology, Kit #9915) kit to confirm cellular apoptosis. Briefly, approximately 0.5 X10^6^ cells were incubated in 6 well plates under various culturing pH and pO_2_ conditions as described above. Aliquots of the cells were collected, and cell numbers were counted before being processed for immunoblot analysis of cleaved caspase 3, or cleaved PARP expression. Cell growth was measured using an WST-1 assay kit (Abcam), which is based on the cleavage of the tetrazolium salt to formazan by cellular mitochondrial dehydrogenase. The amount of the dye generated by activity of dehydrogenase is directly proportional to the number of living cells.

### Immunoblots

Immunoblots were performed using standard procedures. Briefly, cells were lysed in 50 mm Tris pH 7.4, 150 mm NaCl, 1% Nonidet P-40, 1 mm Na3VO4, 10 mm NaF, 2 mm PMSF, 0.5 mm EDTA, 10 μg/ml leupeptin and 10 μg/ml aprotinin. Samples were resolved by 10% SDS-PAGE, transferred to PVDF (0.2 μm) membranes, and probed with antibodies to the following proteins: HIF1α antibody (clone 54/HIF1) was purchased from BD Biosciences (Franklin Lakes, NJ). β-tubulin (clone H-235), goat anti-mouse and goat anti-rabbit IgG horseradish peroxidase-conjugated antibodies were purchased from Santa Cruz Biotechnology (Dallas, TX). The PARP (clone 46D11), caspase 3 (clone D3R6Y), cleaved caspase 3 (clone ASP175), kits were purchased from Cell Signaling Technology (Danvers, MA). The PHD2/EGLN1 (rabbit polyclonal), PHD3/EGLN3 (mouse polyclonal), a PHD3 positive control (PHD3 over expressing lysate from HEK293T cells), and HIF2α (clone ep190b rabbit polyclonal) were purchased from NOVUS Biologicals (Littleton, CO). The carbonic anhydrase CA2 (rabbit polyclonal), CA12 (rabbit polyclonal), MCT-1 (rabbit polyclonal) and MCT-4 (rabbit polyclonal) were purchased from Proteintech (Rosemont, IL). The CA9 (clone 2D3, mouse polyclonal) and LDH (rabbit polyclonal) antibodies were purchased from Novus Biotech (Centennial, CO). All antibodies were used at a final dilution of 1:10,000. Blots were then incubated in appropriate horseradish peroxidase-conjugated secondary antibody and then detected using ECL plus (Amersham). Quantification of hypoxia-mediated changes in protein expression was determined by Densiometric analysis of scanned immunoblots was performed using ImageJ software and is presented as relative change in the expression of proteins of normoxic conditions compared to hypoxic conditions. Briefly the optical density (OD) of the immunoblot bands was measured; the background OD values were subtracted and the data was normalized using loading control actin bands. The relative change was quantified using the formula [OD_normoxia_/OD_hypoxia_] and is expressed as the Mean ± 1 STD of at least 3 independent experiments.

### Animals

Male NSG mouse (NOD.Cg-*Prkdc^scid^ Il2rg^tm1Wjl^*/SzJ) (4-6 weeks old) were obtained from Jackson Laboratories (Bar Harbor, ME). NSG mice lack mature T-cells, B-cells and NK cells as well as being deficient in multiple cytokine signaling pathways and are one of the most immunodeficient model available for xenograft studies. Animal studies were conducted in accordance with protocols approved by the Institutional Animal Care and Use Committee (IACUC) of the Greater Los Angeles Veterans Administration Healthcare System (GLA-VAHS). The mice were maintained in sterile/pathogen free conditions and allowed to acclimatize for 7 days prior to challenge with 8226 human myeloma cell line.

### Subcutaneous xenograft model

8226 cells (10 X 10^6^ cells/mouse) were admixed with matrigel (BD biosciences) then injected subcutaneously into the right flank (200 μl/mouse) of the mice as previously described (11, 17). The mice were monitored daily and randomized into drug treated or control groups (5 mice/group) when the tumor volume reached approximately 300-500 mm^3^. For drug treatments, mice were given intraperitoneal (IP) injections (200 μL/injection) every other day for a total of 4 injections of the CAIX inhibitor, acetazolamide (40 g/kg mouse) or vehicle control at the indicated concentrations. The tumor volume was measured using calipers (width and length) and the tumor volume was calculated using the formula W^2^ X (L/2). In other experiments, animals with SQ tumors were further analyzed using a SOFIA G8 (Culver City, CA) positron emission tomography (PET)/computational tomography (CT) platform for determination of hypoxia *in vivo*.

### [^18^F]-fluoromisonidazole PET/CT experiment

The radiosynthesis of [^18^F]-FMISO, a marker for hypoxia, was carried out according to the 2-step procedure reported by (18) at the Greater Los Angeles VA cyclotron. The total synthesis time was ≈120 min, and radiochemical purity was >99% as assayed by HPLC. Specific radioactivity obtained immediately after the synthesis was >100 GBq/μmol. Animals with palpable tumors measuring approximately 300-500 mm^3^ (approximately day 15-20 days post inoculation) were anesthetized with isoflurane and injected with 5–20 MBq of the radiotracer (100–120 μL per injection) via a lateral tail vein. PET/CT data were acquired in list-mode at 60 min after injection and reconstructed in a single time frame with a voxel size of 1 mm and a matrix size of 120 × 120 × 200 mm. Regions of interest (ROIs) were manually defined by using the dedicated software AMIDE (https://amide.sourceforge.net/). ROIs were drawn for the whole tumor on all coronal planes containing tumor tissue yielding a volume of interest. Reference tissue ROIs were drawn on 5–10 subsequent coronal planes containing muscle tissue at the contralateral side of the animal. The quantification of [^18^F]-FMISO uptake was based on the target-to-muscle retention ratio (TMRR). This ratio was calculated for all hypoxic tumor voxels, with TMRR ≥1.4 defining the presence of hypoxia, analogous to the method described by Koh et al. (19). Additionally, the fractional hypoxic volume of the tumors was computed that is represented by the ratio of hypoxic voxels (with a TMRR ≥1.4) to all voxels within a tumor volume of interest (VOI). For visual inspection and comparison of [^18^F]-FMISO tumor uptake, PET images were normalized to the injected dose per g of body weight. Following individual PET/CT analysis, the animals were returned to their cages and observed until they totally recovered from the anesthesia, kept overnight (for approximately 10 half-lives of ^18^F) and then returned to their home housing room in the GLA Vivarium. This procedure was repeated every 7 days for a total of 3 imaging events/mouse (i.e., one imaging/week for 3 weeks).

### Morphometric analysis

IHC analysis was performed on tissue sections with a Nikon Microphot-SA microscope (Melville, NY) equipped with plan apochromat lenses (20X and 40X). A diagnostic technologies digital camera, model SPOT-RT (Sony), was used to capture images. Final images for publication were prepared using Adobe Photoshop software.

### Statistics

Data was screened for consistency and quality by both graphical (histograms, scatter plots) and analytical methods (descriptive statistics). Variables and pairwise comparisons between treated and control groups were analyzed using generalized linear models (GLM) such as ANOVA and t-tests. Growth curves were assayed using non-parametric regression analysis was used.

## Results

**Figure 1:**
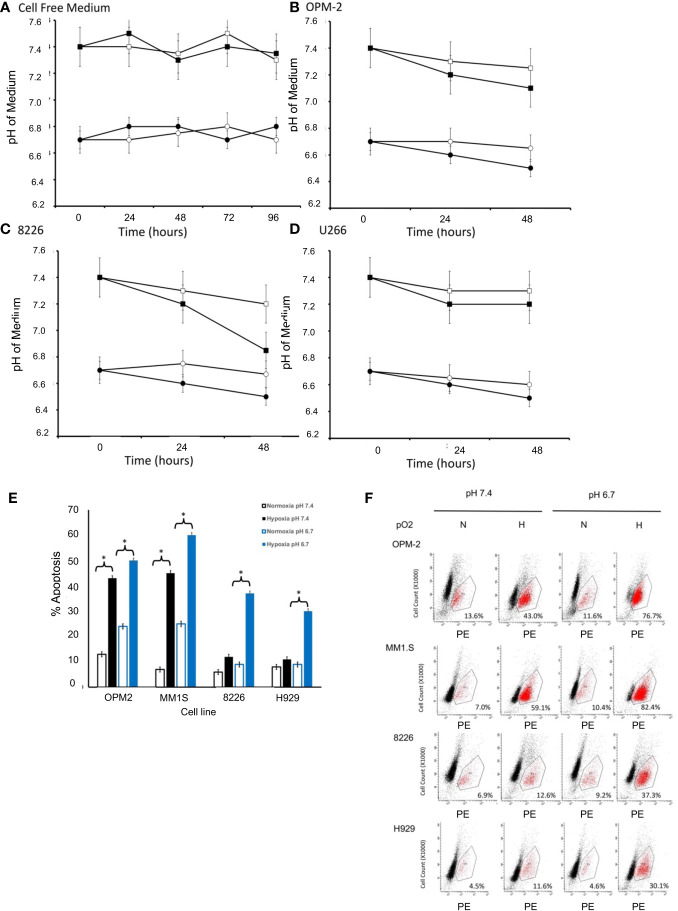
Effects of pO_2_ on pH in cell free modified RPMI-1640 medium (pH 6.7 or 7.4). **(A)** The pH of cell free medium was measured once per day (using a pH electrode). Briefly, modified RPMI-1640 medium was prepared at the indicated starting pH (either 6.7 or 7.4), maintained in standard T75 culturing flasks (25 ml) and cultured in a standard humidified 5% CO_2_ incubator (at 37°C and 22% O_2_) or a dedicated glovebox-design humidified 5% CO_2_ hypoxia chamber (at 37°C and 0.2% O_2_). At the indicated times, 1 ml aliquots of the medium (N=3 samples/time point) were collected and the pH measured using a standard pH electrode (data are presented and mean ± SEM). Squares indicate initial pH of medium at 7.4 and circles indicate initial pH of medium at 6.7, with closed symbols representing normoxic conditions (pO_2 = _22%) and open symbols representing hypoxic conditions (pO_2 = _0.2%). Effects of pO_2_ on pH in cell culture. Briefly, 1X10^6^ cells were cultured in standard T75 culturing flasks (in 25 ml) in pH modified RPMI-1640 medium (6.7 or 7.4) and maintained in either a standard humidified 5% CO_2_ incubator or a dedicated glovebox-design humidified hypoxia chamber as described above. Aliquots of the medium (N=3 samples/time point) were collected at various times and the pH measured using a standard pH electrode (Data are presented and mean ± SEM). Squares indicate initial pH of medium at 7.4 and circles indicate initial pH of medium at 6.7, with closed symbols representing normoxic conditions (pO_2 = _22%) and open symbols representing hypoxic conditions (pO_2 = _0.2%). Changes in pH over time are shown for **(B)** OPM-2 cells. **(C)** 8226 cells. **(D)** U266 cells. Cytotoxic effects of pO_2_ and pH on various human MM cell lines cultured in modified RPMI-1640 medium **(E)** The % apoptosis in MM cells (OPM2, MM1S, 8226, H929 cells) was measured in cells (1X10^6^ cells/ml) that had been cultured as described above and changes in levels of cleaved caspase 3 expression was measured by flow cytometry using a cleaved caspase 3 kit. The cells were maintained in pH modified medium (either 6.7 or 7.4) under “normoxic” conditions (~22% O_2_) or hypoxic (0.2% O_2_) conditions. Values are presented as means with 95% CI error bars of 4 independent experiments/cell line. Flowcytometry histograms for cleaved caspase 3 expression in cells cultured as described above. **(F)** Representative histograms for cell death of MM cell lines. The values in the lower right quadrant indicate the percent of cells that were positive for PE-labeled cleaved caspase expression within the gated region.

Mammalian cell lines are typically cultured in media formulated with various buffering reagents (e.g. HEPES, TRIS, NaHCO_3_) designed to maintain the pH within a specific physiologic range (usually around a pH of 7.0-7.4) in a 37°C, humidified atmosphere at 22% O_2_ and 5% CO_2_ (16). While these conditions are the standards for cell culture *in vitro*, our modified culturing conditions are more relevant to the actual physiological TME that exists *in vivo* (lower hypoxic and lower pH conditions). First, we established that the pH of our cell-free modified medium was stable and did not change when cultured under normoxic or hypoxic conditions in 5% CO_2_ and a humidified 37°C environment (measured daily up to 96 hours) (**Figure 1A**). Since growing cells acidify their culturing medium over time due to the buildup of acidic metabolic byproducts, we next examined the changes in pH induced by growing cells in our modified medium at different levels of pO_2_. As seen in **Figures 1B–D**, the test medium tended to become slightly more acidic (a drop of about 0.2-0.5 pH) overtime, however, the relative differences between the two modified pH formulations were similar over time for both the hypoxia-sensitive OPM2 cell line (**Figure 1B**), as well as for the hypoxia-resistant 8226 (**Figure 1C**) and U266 (**Figure 1D**) cell lines.

### Effects of low pO_2_ and pH on survival of MM tumor cells

We have previously shown that 8226, U266 and H929 MM cell lines are resistant to low pO_2_, while OPM2 and MM1S are more sensitive to hypoxia-mediated apoptosis (12, 13, 20). What remains unclear is whether this is also correlated to sensitivity to an acidic pH. Therefore, we asked if these MM cell lines demonstrated a similar pattern of sensitivity to killing when cultured in low pH conditions. As shown in **Figure 1E**, As expected, under baseline conditions (normoxic and control medium (pH 7.4)), little killing (>90% survival) of cultured MM cells was observed. However, normoxic pO_2_ and an acidic (pH 6.7) induced a modest but significant increase in cell death for the hypoxia-sensitive OPM2 and MMIS cells (~ 20-25% death) while the hypoxia-resistant 8226 and H929 lines were less sensitive (~10-15% death) (ANOVA * and bracket indicates p<0.05 of treatment versus control). Furthermore, culturing the cells under hypoxic conditions (0.2%) induced a significantly higher levels of apoptosis (43% for OPM2 and 45% for MMIS respectively) in control medium (pH 7.4) and this was further augmented under acidic conditions (~ 60% killing for both cell lines). In comparison, 8226 and H929 (as well as U266, data not shown) were less sensitive to hypoxia and acidic culturing conditions, with significantly less killing (~30-35%) over the baseline. **Figure 1F** shows representative histograms for cleaved caspase 3 flow cytometry data demonstrating sensitivity to apoptosis of the various cell lines under hypoxic and acidic culturing conditions as described above.

### Sensitivity to pH is correlated to HIF expression

**Figure 2:**
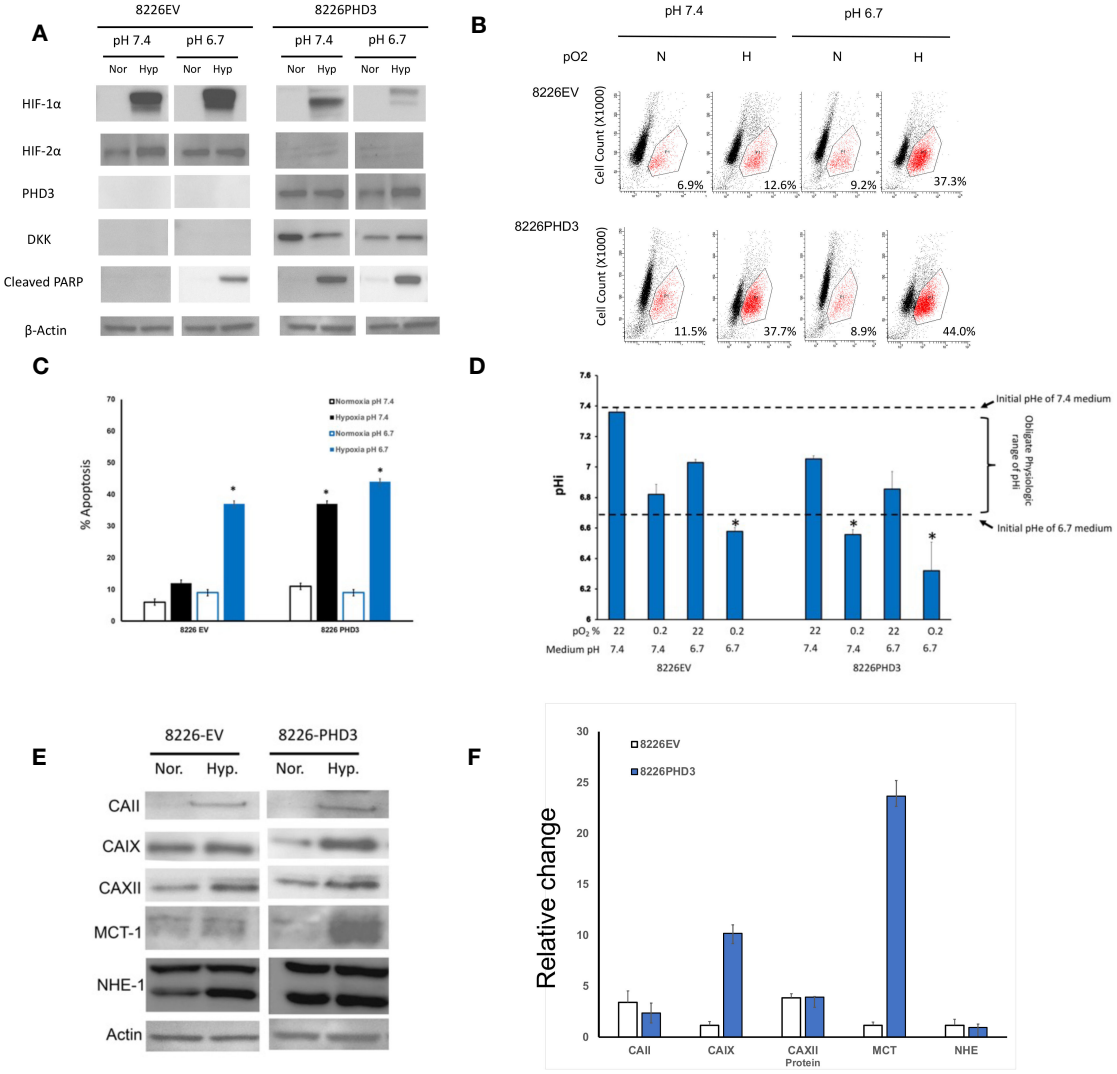
Characterization of 8226EV and 8226PHD3 MM cells **(A)** Immunoblots of 8226EV and 8226PHD3 cells cultured under normoxic or hypoxic conditions. Briefly, cells were cultured in modified RPMI-1640 under various pO_2_ levels then harvested on ice and the lysate was immunoblotted for HIF1α and HIF2α and PHD3. Cellular apoptosis was measured by expression of the apoptosis markers, PARP. Exogenous expression of the transduced PHD3 allele was confirmed by expression of DKK tag. Exogenous expression of PHD3 sensitizes 8226 cells to low pO_2_- and pH-mediated apoptosis. **(B)** Representative flowcytometry histograms of cleaved caspase 3 expression in cells cultured under various pO_2_ and pH levels as described above [similar results were observed using Annexin V (see **Supplemental Data**)]. The values in the lower right quadrant indicate the percent of cells that were positive for PE-labeled cleaved caspase expression. **(C)** Quantification of flowcytometry data (data was compiled from 3 independent experiments). Values are mean ± 1 STD of percent of cleaved caspase 3 positive cells. * Indicate p<0.05. PHD3 expression is correlated with acidic pHi. **(D)** Analysis of pH values in the external (medium) pHe compartment and internal (intracellular) pHi compartment of cells cultured under various pO_2_ and pH conditions. The pHi was measured using a fluorometric intracellular pH kit, and the extracellular pHe was measured using pH electrode. The dashed lines indicate the baseline medium pH (either 7.4 or 6.7) and the bracket indicates the presumptive “obligate” level of pHi required for cell survival. Those values of pHi that are marked with * that indicate treatments that correspond to significant apoptosis in cell culture (see **C**). Exogenous expression of PHD3 affects expression of pH regulating factors in 8226 cells. **(E)** Immunoblots of pH regulating factors in 8226EV and 8226PHD3 cells that were cultured under conditions as described above (data shown are representative immunoblot of 3 independent experiments. **(F)** Densitometric analysis of relative change in protein expression under normoxic conditions compared to hypoxic conditions is presented in the bar graph (right panel). Data represents the mean ± STD of at least 3 experiments. * Indicate p<0.05.

We have previously reported that the phenotype of hypoxia-resistant 8226 cells is reversed by using siRNA to knock out the expression of HIFα subunits (see (13) and **Supplemental figure 1A**). Since HIF2α was constitutively expressed we focused on altering the exogenous expression of prolyl-4-hydroxylase domain-3 (PHD3), a presumptive tumor suppressor gene and the primary regulator of HIF2α degradation (21). **Figure 2A** [and previously reported by us (13, 20)], demonstrates that the endogenous PHD3 gene is silenced in parental 8226 cells. To study the role of PHD3 in 8226 cells, we have previously generated isogenic 8226 cell lines transduced with a PHD3 allele or empty vector control (this gene has a DKK tag). The parental 8226 cell line [data not shown but previously reported (13, 20)] and 8226EV lack endogenous PHD3 and in its absence, HIF2α is constitutively expressed (**Figure 2A**). On the other hand, the expression of HIF1α is absent under normoxia conditions but is strongly upregulated in hypoxic conditions (**Figure 2A**). In comparison, 8226PHD3 expression was correlated to the down regulated expression of HIF2α. The 8226PHD3 cells also demonstrated significantly increased hypoxia-mediated apoptosis compared to isogenic 8226EV control cells as assayed by immunoblot for cleaved PARP (**Figure 2A**), by flow cytometry for cleaved caspase 3 (**Figure 2B**; **Supplemental figure 2A**) and for altered growth measured by WST-1 (**Supplemental figure 1B**). Similar results were found using an Annexin V/PI kit [see **Supplemental figure 2B** and (13, 20)]. **Figure 2C** presents a summary of flow cytometry data (results from 4 independent experiments) and demonstrates a significant sensitization of 8226PHD3 cells to hypoxia-mediated killing (p<0.05) (an increase from 11% to 37% killing) when compared to 8226EV control cells. Furthermore, low pO_2_-mediated killing was significantly greater in cells cultured in acidic (pH 6.7) medium compared to cells cultured in basic/neutral (pH 7.4 medium).

### Hypoxia-mediated cell death is correlated to an acidic intracellular pH

The relationship of pH on cell growth is complex as there are distinct intracellular (pHi) and extracellular pH (pHe) compartments. Maintaining the pHi within a specific range is necessary for the proper function of enzymes in the cytoplasm and is an obligate requirement for cell survival. As shown in **Figure 2D**, we compared the change in pHe (open bars) and the pHi (solid bars) compartments in the hypoxia-resistant 8226EV and hypoxia-sensitive 8226PHD3 cell lines cultured under various conditions. The pHe of 8226EV and 8226PHD3 cells grown under normoxic conditions closely reflect the baseline values of the modified medium’s initial pH (dashed lines at 7.4 and 6.7 respectively). Furthermore, the pHi of 8226EV cells grown under normoxic (in 6.7 or 7.4 medium) or hypoxic conditions (in 7.4 medium) were seen to be maintained within the presumed obligate pHi physiological range (see brackets) (an approximate pHi of 6.8-7.4). The exception to this were 8226EV cells cultured in acidic medium and under hypoxic conditions where the pHi was measured to be less than < 6.7 (marked as *). In contrast to 8226EV control cells, the pHi of 8226PHD3 cells grown under hypoxic conditions (in pHe 6.7 or 7.4 medium) was below this physiological range of values (pHi 6.7 and indicated by *). This suggested that there was a correlation between acidic pHi below “obligate” levels and hypoxia-mediated cell death. We next compared the changes in a panel of pH-regulating proteins in isogenic 8226 cells (**Figure 2E**). Several factors, like CAII, CAIX, and MCT were upregulated in 8226PHD3 cells cultured under hypoxic and acidic conditions (**Figure 2E** shows representative Immunoblots of at least 3 independent experiments. The relative change in levels of protein expression in cells cultured under hypoxic and acidic conditions was quantified by densitometry (**Figure 2F**). The values are presented as the average relative change (± 1STDEV) in expression of each factor for hypoxic culturing conditions compared to normoxic conditions (values >1 indicate upregulation by hypoxia).

### Effects of hypoxia on pHe and pHi

**Figure 3:**
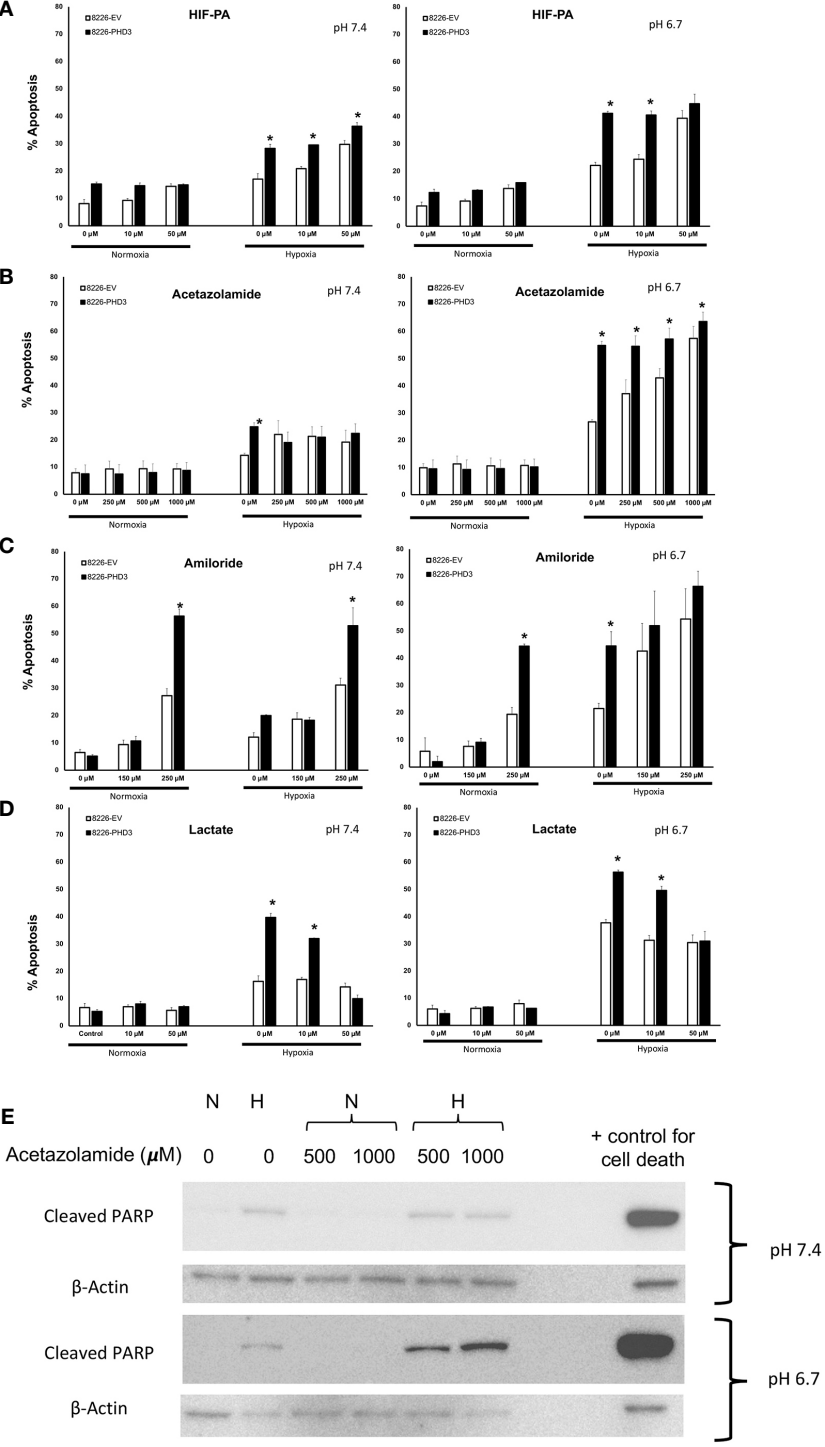
Inhibition of pH regulatory pathways sensitizes 8226 MM cells to apoptosis under low pO_2_/pH conditions that is dependent on exogenous expression of PHD. Effects of hypoxia- and pH-mediated killing on isogenic 8226 cell lines (8226EV = open bars; 8226PHD3 = closed bars) treated with various HIF or pH regulating drugs. **(A)** Acetazolamide treatment sensitizes isogenic 8226 cell lines (8226EV = open bars; 8226PHD3 = closed bars) to hypoxic and acidic pH-mediated killing. **(B)** Amiloride treatment sensitizes isogenic 8226 cell lines to hypoxic and acidic pH-mediated killing. **(C)** HIF-PA treatment sensitizes isogenic 8226 cell lines to hypoxic and acidic pH-mediated killing. **(D)** Na-lactate treatment rescues isogenic 8226 cells to hypoxia- and low pH-mediated killing. Cells were cultured as described above. Cell death was determined by flow cytometry assay for cleaved caspase 3, and the values represent the mean ± 1 STD of 3 independent experiments. *, p<0.05 (T-test EV compared to PD3 cells for indicated treatments). **(E)** Immunoblot analysis of cleaved PARP in Na-lactate treated isogenic 8226 cells cultured at pH 6.7. Cells were treated with indicated concentrations of Na-lactate for 48 hours. Staurasporin (1uM) was used as a positive control for apoptosis.

Our previous studies demonstrated that PHD3 and HIF regulate the sensitivity to hypoxia-mediated killing in MM cells *in vitro* (13, 20). Because acidic pHe is often correlated with low pO_2_ levels we hypothesized that HIF-mediated regulation of pH is critical for cellular survival. To test this hypothesis, we used the sequence-specific DNA-binding pyrrole-imidazole (Py-Im) polyamide, which disrupts the HIF (HIF-PA) heterodimer from binding to its cognate DNA sequences (12) (**Figure 3A**). We also used an inhibitor of carbonic anhydrase 9 (acetazolamide) (**Figure 3B**), or an inhibitor of NHE-1 (amiloride) (**Figure 3C**). Both drugs were selected because they are known to inhibit the ability of cells to regulate intracellular pH. As shown in **Figure 3A–C**, all three inhibitors were more effective at killing 8226PHD3 (when compared to empty vector control cells) under hypoxic conditions, and these effects were more pronounced when the cells were cultured in acidic medium. To further explore the role of intracellular pH, we also cultured cells in Na-lactate (**Figure 3D**), which is a sodium salt with alkalinizing properties. Upon metabolism, Na-lactate is converted to bicarbonate, which facilitates removal of hydrogen ions and lactate and leads to an increase in pH. As shown in **Figure 3D**, treatment with Na-lactate protected the cells from hypoxia-mediated killing as well as an increase in pHi. We confirmed that acetazolamide induced apoptosis under hypoxic and low pH condition by immunoblot for cleaved PARP (**Figure 3E**).

### Hypoxia-mediated cell death is correlated to an acidic pHi below obligate levels

**Figure 4:**
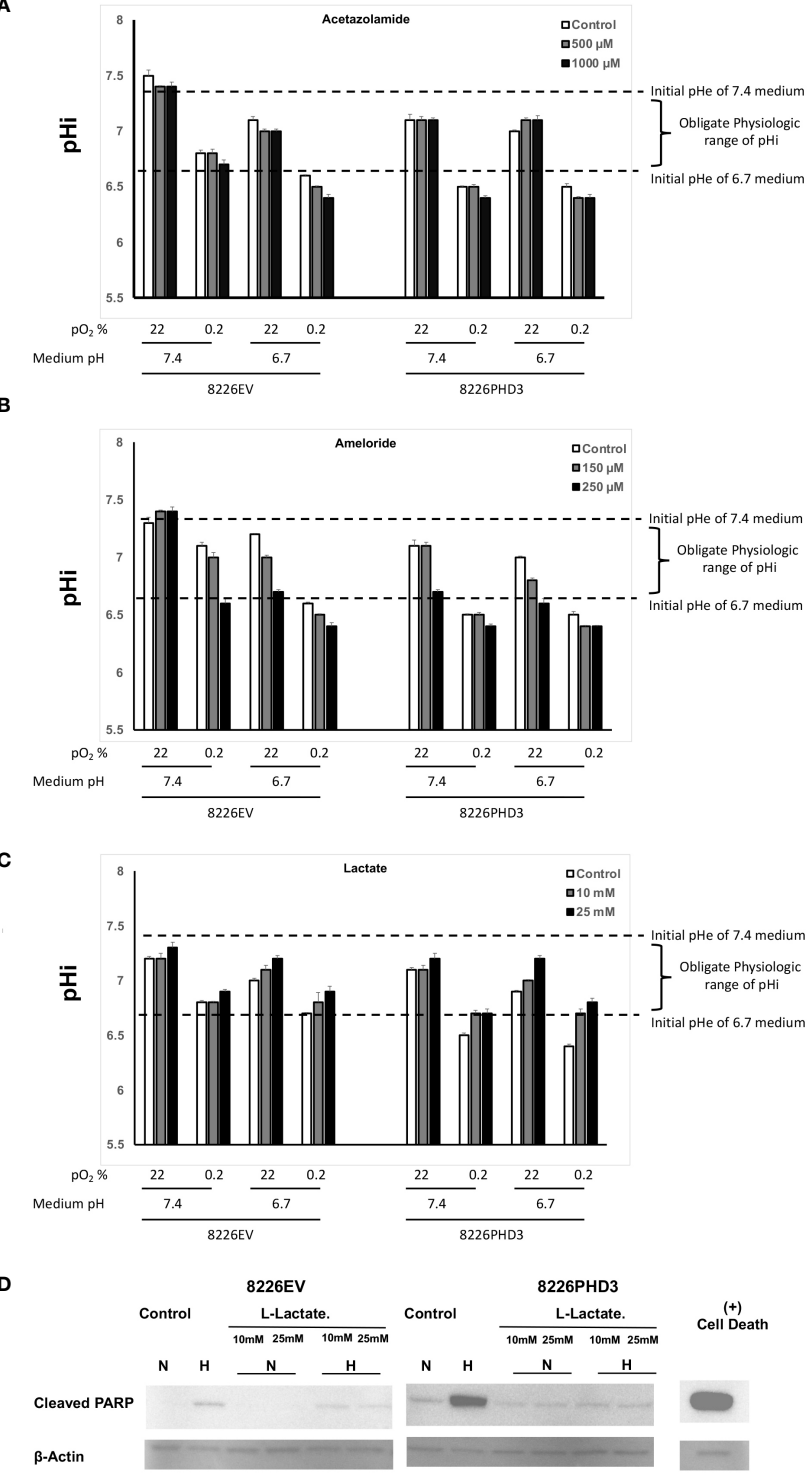
Changes in pHi and pHe in isogenic 8226 cells. **(A)** Effect of acetazolamide on the external (medium) pHe compartment and internal (intracellular) pHi compartment of isogenic 8226 cells cultured under various pO_2_ and pH conditions. The pHi was measured using a fluorometric intracellular pH kit, and the extracellular pHe was measured using a pH electrode. The dashed lines indicate the baseline medium pH (either 7.4 or 6.7) and the bracket indicates the presumptive “obligate” level of pHi required for cell survival. **(B)** Effect of amiloride on the external (medium) pHe compartment and internal (intracellular) pHi compartment of isogenic 8226 cells cultured under various pO_2_ and pH conditions. The pHi was measured using a fluorometric intracellular pH kit, and the extracellular pHe was measured using a pH electrode. The dashed lines indicate the baseline medium pH (either 7.4 or 6.7) and the bracket indicates the presumptive “obligate” level of pHi required for cell survival. **(C)** Effect of Na-lactate on the external (medium) pHe compartment and internal (intracellular) pHi compartment of isogenic 8226 cells cultured under various pO_2_ and pH conditions. The pHi was measured using a fluorometric intracellular pH kit, and the extracellular pHe was measured using a pH electrode. The dashed lines indicate the baseline medium pH (either 7.4 or 6.7) and the bracket indicates the presumptive “obligate” level of pHi required for cell survival. **(D)** Immunoblot analysis of cleaved PARP in Na-lactate treated isogenic 8226 cells.

As seen in **Figure 4A**, the pH_i_ in isogenic 8226 cells cultured in hypoxic and acidic conditions showed a significant decrease in the pH from ~pH 6.6 to pH 6.4 following treatment with acetazolamide and a similar effect was observed in cells treated with amiloride (**Figure 4B**). These ranges of pHi fell below the “obligate levels” of pHi and are marked by an “*”. In both cases the treatment also resulted in a decreased pHi in cells cultured under hypoxic conditions. The acidification of the pHi in both acetazolamide and amiloride treated cells correlated closely with increased hypoxia- and acidic pH-mediated apoptosis (compare to **Figures 3B, C** respectively). We also observed a protective effect on pHi in Na-lactate treated cells (**Figure 4C**) which also correlated with decreased sensitivity to hypoxia-mediated apoptosis (compare to **Figure 3D**). We confirmed that Na-lactate protected cells from apoptosis cultured under hypoxic and low pH (pH 6.7) conditions by immunoblot for cleaved PARP (**Figure 4D**).

### Acetazolamide kills MM tumors *in vivo*

**Figure 5:**
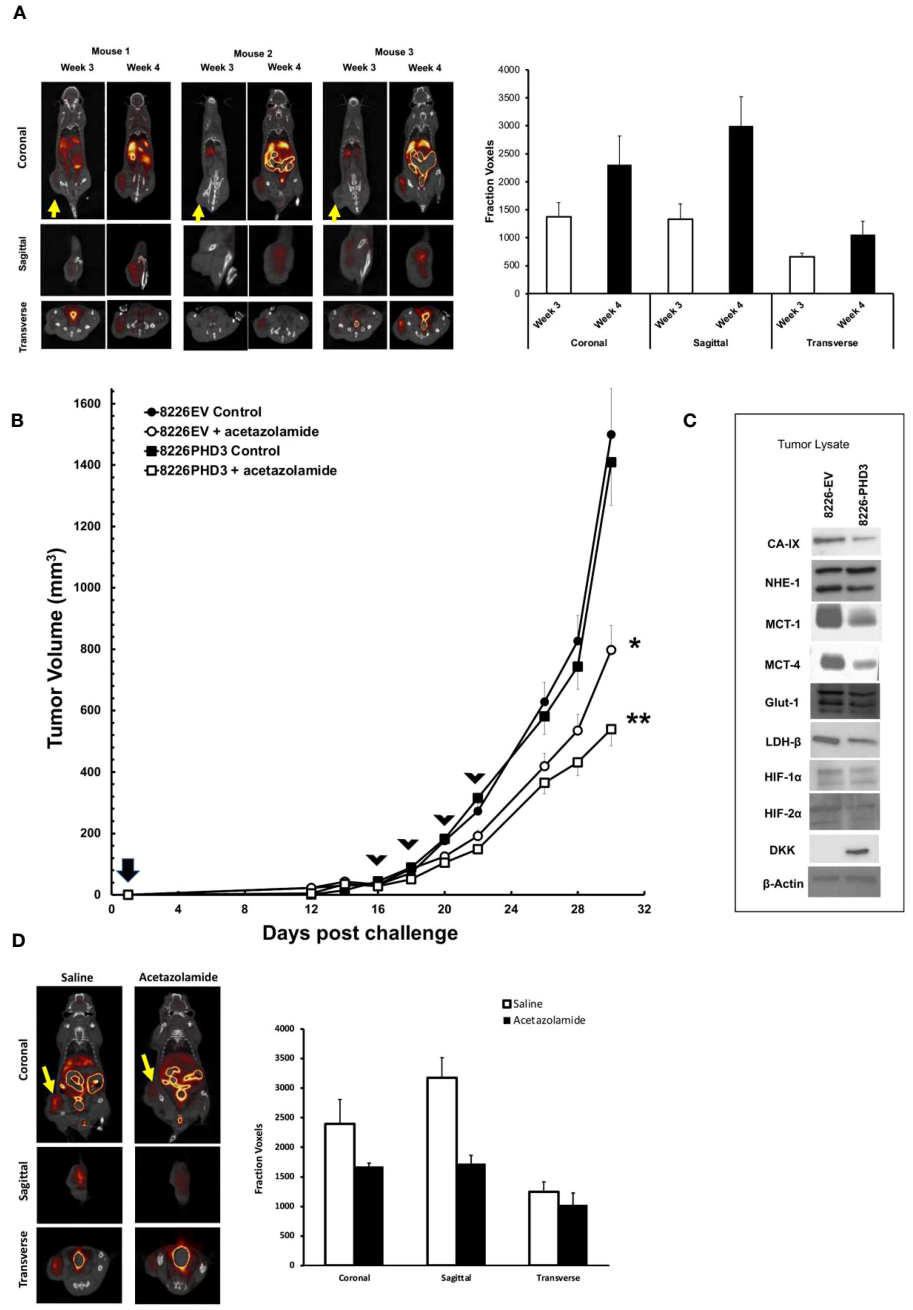
8226 tumor growth induces hypoxia measured by ^18^F-FMISO hypoxia probe. **(A)** Uptake of ^18^F-FMISO probe determined by PET/CT analysis of 3 SQ 8226 tumors grown in 3 individual mice (out of 4 total mice). Mice were challenged at day 0, and images were collected at day 21 (3 weeks) and day 28 (4 weeks) using PET/CT. Yellow arrow indicates location of SQ tumor. Coronal, sagittal and transverse images of the tumors are shown. Right panel. Summary of ^18^F-FMISO uptake (presented as mean ± 1 STD voxels) at coronal, sagittal and transverse layers of tumor (N=4 mice) at day 21 (3 weeks) and day 28 (4 weeks). **(B)** Effects of acetazolamide treatment on growth of isogenic 8226 tumors in mice (N=4). Mice were challenged on day 0 with isogenic 8226 tumor cell lines (8226EV = open symbols; 8226PHD3 = closed symbols) and monitored daily for tumor growth. On day 12 post challenge, the tumors were large enough to be measured using calipers, and the volume was determined. IP injections of acetazolamide (40 μg/Kg mouse in saline) or control were administered on days 16, 19, 21, and 23 (indicated by arrow heads). The changes in tumor volume are plotted over time. Statistical analysis by non-parametric analysis of the growth curves; * p<0.05 of acetazolamide treated 8226EV tumors versus untreated 8226EV tumors. ** p<0.05 of acetazolamide treated 8226EV tumors versus acetazolamide treated 8226PHD3 tumors **(C)**. At conclusion of the experiment, the mice were humanely sacrificed, and the tumor nodules collected (N=4), flash frozen and then processed for immunoblot analysis for indicated proteins. **(D)** Effects of acetazolamide on ^18^F-FMISO uptake was assayed by PET/CT analysis. Mice (N=4) were challenged with 8226 cells and treated with acetazolamide or vehicle control on day 16, 19, 21, and 23 as described above. Left panel shows representative mice images on day 28l. Right panel shows summary of 18F-FMISO uptake (presented as mean ± 1 STD voxels) at coronal, sagittal and transverse layers of tumor (N=4 mice) at day 28 (4 weeks).

Because *in vitro* culturing conditions may not accurately reflect the *in vivo* effects of hypoxia in tumor nodules, we used an SQ xenograft model of MM tumors and PET/CT imaging technology to study hypoxia within tumor nodules (**Figure 5**). We previously demonstrated that xenograft subcutaneous MM tumors develop regions of hypoxia and this colocalized to regions of cellular apoptosis (12). As shown in **Figure 5A**, analysis of SQ 8226 tumors labeled with ^18^F-FMISO, a marker of hypoxia, showed a punctate distribution of probe uptake that could be observed by PET/CT (**Figure 5A** left panel—shows whole mouse uptake of ^18^F-FMISO, yellow arrow shows localization of tumor nodule). The right panel of **Figure 5A** shows the summary analysis of ^18^F-FMISO increased uptake in tumors over time. We next tested if acetazolamide inhibited tumor growth. Mice (N=6/group) were given SQ challenge with either 8226EV or 8226PHD3 cells and once the tumor reached ~200 mm^3^ they were randomized and given 4 IP injections of acetazolamide (40 μg/Kg mouse) or vehicle control on days 16, 18, 22 and 24. As shown in **Figure 5B** we observed a statistically significant inhibition of tumor growth in treated mice (*= p<0.05 in treated 8226EV tumors) starting after the third treatment and these effects were greater in the 8226PHD3 tumors (** p<0.05). At the end of the experiment, we excised tumor nodules, and the lysate was immunoblotted for various acid/base regulating proteins, such as CAIX, MCT-1 and MCT-4 that were all downregulated in 8226PHD3 tumors compared to their isogenic control 8226EV cells (**Figure 5C**). Finally, we also observed a significant effect of ^18^F-FMISO uptake in tumors in acetazolamide (40 μg/Kg mouse) treated mice as measured by PET/CT (**Figure 5D** left panel and quantified in right panel).

### Discussion

Targeting the hypoxic response in the TME as a mode for killing tumor cells has been well explored for a variety of anti-cancer mechanisms and strategies (such as inhibiting angiogenesis or HIF activity) [for review see (22)]. Physiologic pO_2_ in “normal” tissues can vary over time and is based on the tissue vasculature and cellular metabolic needs, which is organ specific and can vary broadly from 40-85 mmHg (23). Some tissues can exhibit even lower pO_2_ levels, such as the BM, which was found to range from 10-30 mmHg (and even lower in some interstitial spaces) (3). Thus, it remains unclear as to what specific “hypoxic” threshold (other than total anoxia) is cytotoxic to tumor cells, and whether this can be leveraged for anti-tumor therapy. In fact, there is a growing consensus that the opposite effect occurs, in which low pO_2_ levels are strongly correlated with the development of more aggressive and malignant tumor phenotypes. The fact that many cells (both normal and malignant) have developed effective and robust pro-survival and pro-growth responses to hypoxia, such as activation of HIF inducible factors, suggests that the more effective target would be identifying and inhibiting those critical adaptive hypoxic responses (12).

There are several physiologic conditions that impact tumor cell survival under conditions of low pO_2_, including changes in pH that are due to alterations in aerobic and anaerobic metabolic pathways (the Warburg effect) (24). One of these adaptive cellular hypoxic response mechanisms is the requirement that pHi is maintained at obligate levels for cell survival (seen as the development of a “reverse” pH gradient in which the extracellular space is more acidic than the intracellular space. In this study, we observed that myeloma cells were relatively resistant to hypoxia-mediated apoptosis (**Figure 2**) and extracellular acidic pH, but when the cells were co-cultured at low pO_2_ conditions (0.2%) and in acidic (pH 6.7) medium, there was a significant sensitization of hypoxia-mediated cell death. The fact that very low hypoxic conditions (down to 0.2%) was insufficient to kill MM cells suggested that the underlying factor involved in sensitivity was related to the inability of cells to maintain the pH within those obligate levels that are required for survival (~pHi 6.7-7.2).

The master regulator of the hypoxic adaptive response is mediated, at least in part, by the transcriptional factor HIF, which consists of constitutively expressed β-subunit (HIFβ) and inducible α-subunits (e.g. HIF1, 2, or 3α) that are frequently upregulated in MM patients (25). The O_2_-dependent proteasome degradation of HIF is mediated by several PHD proteins (including PHD3). In the absence of O_2_, the stability of the HIFα-subunit increases which allows for dimerization to the HIFβ-subunit, leading to nuclear localization of hypoxia response elements (HREs). We recently reported that PHD3 is downregulated in the hypoxia-resistant 8226 MM cell line and restoring its expression rescued O_2_-dependent regulation of HIF2α, resulting in hypoxia-mediated apoptosis (13). This sensitivity to low pO_2_ was significantly increased when the cells were cultured in acidic medium (pH ~6.7) compared to a medium with a neutral/basic pH (**Figures 3A–C**). The pH balance within cells is very complex, and is carefully regulated within the various cellular compartments (i.e. mitochondria, lysosomes, nucleus, cytoplasm), ranging in pH from about 4-8, depending on the organelles and their function. It remains critical that the pHi of these compartments is maintained, and any failure to maintain the pHi results in cell death. Thus, while cancer cells typically utilize more anaerobic metabolic pathways and tend to excrete significantly higher levels of acidic byproducts, they still need to maintain their intracellular pH at these obligate levels.

We previously reported that hypoxia upregulates various acid/base regulating factors through the activity of HIF and PHD3 in 8226 cells (13, 20). Furthermore, we also showed that the CAIX inhibitor, acetazolamide, could sensitize cells to hypoxia-mediated cell death (13, 20). CAIX is a membrane bound protein that plays a crucial role in maintaining intracellular pH within a neutral/alkaline range under hypoxic conditions (26). It was recently reported that the sera of patients with relapsed/progressed MM reacted with antibodies to CAI, II, IX and XII and patients with elevated CA autoantibody titers had a significant survival benefit over those who did not (27). Importantly, inhibition of CAIX activity leads to reduced tumor growth and inhibition of metastases, as well as depletion of cancer stem cell populations when used against various hypoxic tumors (28, 29). We found that targeting pH using a HIF inhibitor (HIFPA), CAIX inhibitor (acetazolamide) and NHE-1 inhibitor (amiloride) sensitized the hypoxia-resistant 8226EV cells to hypoxia-mediated apoptosis This was confirmed using immunoblot for cleaved PARP, a marker for apoptosis. Treating cells with these inhibitors also resulted in an acidified pHi compartment that was indicative of inhibited pH regulation that correlated strongly to increased cell death (**Figure 4C**). On the other hand, using Na-lactate, an alkalinization factor, had the opposite effect and protected the cells. Finally, because the *in vitro* environment may not always accurately represent the tumor microenvironment, we also tested if acetazolamide inhibited the growth of 8226 SQ tumors grown in mice. Our data supported the hypothesis that targeting pH regulation in the tumor nodules can sensitize MM tumor cells to hypoxia induced cell death.

## Conclusion

The changes in cellular pH caused by hypoxia are a critical factor in allowing tumor cells to survive in an inimical environment. Targeting the pH regulation pathways resulted in greater sensitivity to hypoxia-mediated cell death, *in vitro* and *in vivo*. In the BM, hypoxia is a critical underlying component of MM tumor growth, survival, and progression and this appears to be mediated, at least in part, by regulation of the internal cellular pH at obligate levels. Targeting the hypoxia-mediated adaptive mechanisms that help maintain pHi at these critical levels results in significant upregulation of cellular apoptosis.

## Data availability statement

The original contributions presented in the study are included in the article/**Supplementary Material**. Further inquiries can be directed to the corresponding author.

## Ethics statement

Ethical approval was not required for the studies on humans in accordance with the local legislation and institutional requirements because only commercially available established cell lines were used. The animal study was approved by Greater Los Angeles VA Healthcare System IACUC. The study was conducted in accordance with the local legislation and institutional requirements.

## Author contributions

GG: Formal Analysis, Investigation, Writing – original draft, Writing – review and editing. JK: Conceptualization, Funding acquisition, Investigation, Writing – review and editing. MV: Data curation, Formal Analysis, Investigation, Methodology, Supervision, Writing – review and editing. AB: Data curation, Formal Analysis, Investigation, Methodology, Writing – review and editing. CL: Data curation, Investigation, Writing – review and editing. KL: Data curation, Formal Analysis, Investigation, Writing – review and editing. EC: Conceptualization, Methodology, Writing – review and editing. PF: Conceptualization, Data curation, Formal Analysis, Funding acquisition, Investigation, Methodology, Project administration, Resources, Supervision, Validation, Visualization, Writing – original draft, Writing – review and editing.

## Supplementary material

Supplementary Figure 1 Knockdown of HIFα-subunits sensitizes 8226 cells to hypoxia-mediated apoptosis. **(A)** 8226 cells were transfected with HIF1α siRNA, HIF2α siRNA or both HIF1α and HIF2α siRNA. Non-specific scrambled siRNA was used as a control. The cells were then cultured under normoxic (22% O_2_ white bars) or hypoxic (0.1% 0_2_ black bars) conditions for 48 hours and apoptosis was measured by flow cytometry for cleaved caspase-3. The data shows mean ± SEM of 3 independent experiments. **(B)** Hypoxia reduces the growth of 8226PHD3 compared to 8226EV cells and this effect is augmented by acidic pH. WTS-1 assay was used to measure cell growth. Data is presented as mean ± 1 STD. * = p<0.05 (t-Test) for pairwise comparison of 8226EV (clear bars) compared to 8226 (blue bars).

Supplementary Figure 2 Exogenous expression of PHD3 increases sensitivity of 8226 cells to hypoxia-mediated apoptosis under acidic conditions. **(A)** Representative histogram of isogenic 8226 cells cultured under variable pO_2_ and pH conditions 48 hrs. Apoptosis was measured by flow cytometry for cleaved caspase-3. The first set of panels show the forward scatter (FSC) plotted against the side scatter (SSC). The second set of panels shows the histogram of number of cells against the expression of PE and the gate used to differentiate between PE positive and PE negative cell populations. The values represent % apoptosis (PE positive) cells compared to total number of cells. **(B)** Apoptosis was measured in the same cells cultured as describe above, but with an Annexin V/PI apoptosis staining kit.

## References

[1] SEER*Explorer: An interactive website for SEER cancer statistics. Surveillance Research Program, National Cancer Institute (2020). Available at: https://seer.cancer.gov/statistics-network/explorer/.

[2] Cowan AJ Green DJ Kwok M Lee S Coffey DG Holmberg LA. Diagnosis and management of multiple myeloma: A review. JAMA (2022) 327(5):464–77. doi: 10.1001/jama.2022.0003 35103762

[3] Spencer JA Ferraro F Roussakis E Klein A Wu J Runnels JM. Direct measurement of local oxygen concentration in the bone marrow of live animals. Nature (2014) 508(7495):269–73. doi: 10.1038/nature13034 PMC3984353 24590072

[4] Hu J Van Valckenborgh E Menu E De Bruyne E Vanderkerken K. Understanding the hypoxic niche of multiple myeloma: therapeutic implications and contributions of mouse models. Dis Model Mech (2012) 5(6):763–71. doi: 10.1242/dmm.008961 PMC3484859 23115205

[5] Jing X Yang F Shao C Wei K Xie M Shen H. Role of hypoxia in cancer therapy by regulating the tumor microenvironment. Mol Cancer (2019) 18(1):157. doi: 10.1186/s12943-019-1089-9 31711497 PMC6844052

[6] Fais S Venturi G Gatenby B. Microenvironmental acidosis in carcinogenesis and metastases: new strategies in prevention and therapy. Cancer Metastasis Rev (2014) 33(4):1095–108. doi: 10.1007/s10555-014-9531-3 PMC4244550 25376898

[7] Martin SK Diamond P Gronthos S Peet DJ Zannettino AC. The emerging role of hypoxia, HIF-1 and HIF-2 in multiple myeloma. Leukemia (2011) 25(10):1533–42. doi: 10.1038/leu.2011.122 21637285

[8] Maiso P Huynh D Moschetta M Sacco A Aljawai Y Mishima Y. Metabolic signature identifies novel targets for drug resistance in multiple myeloma. Cancer Res (2015) 75(10):2071–82. doi: 10.1158/0008-5472.CAN-14-3400 PMC4433568 25769724

[9] Asosingh K De Raeve H de Ridder M Storme GA Willems A Van Riet I. Role of the hypoxic bone marrow microenvironment in 5T2MM murine myeloma tumor progression. Haematologica (2005) 90(6):810–7. 15951294

[10] Frost P Shi Y Hoang B Lichtenstein A. AKT activity regulates the ability of mTOR inhibitors to prevent angiogenesis and VEGF expression in multiple myeloma cells. Oncogene (2007) 26:2255–62. doi: 10.1038/sj.onc.1210019 17016437

[11] Frost P Berlanger E Mysore V Hoang B Shi Y Gera J. Mammalian target of rapamycin inhibitors induce tumor cell apoptosis in *vivo* primarily by inhibiting VEGF expression and angiogenesis. J Oncol (2013) 2013:897025. doi: 10.1155/2013/897025 23533410 PMC3603547

[12] Mysore VS Szablowski J Dervan PB Frost PJ. A DNA-binding molecule targeting the adaptive hypoxic response in multiple myeloma has potent antitumor activity. Mol Cancer Res (2016) 14(3):253–66. doi: 10.1158/1541-7786.MCR-15-0361 PMC4794370 26801054

[13] Gastelum G Poteshkina A Veena M Artiga E Weckstein G Frost P. Restoration of the prolyl-hydroxylase domain protein-3 oxygen-sensing mechanism is responsible for regulation of HIF2alpha expression and induction of sensitivity of myeloma cells to hypoxia-mediated apoptosis. PloS One (2017) 12(12):e0188438. doi: 10.1371/journal.pone.0188438 29206844 PMC5716583

[14] Aprelikova O Chandramouli GV Wood M Vasselli JR Riss J Maranchie JK. Regulation of HIF prolyl hydroxylases by hypoxia-inducible factors. J Cell Biochem (2004) 92(3):491–501. doi: 10.1002/jcb.20067 15156561

[15] Belitsky JM Nguyen DH Wurtz NR Dervan PB. Solid-phase synthesis of DNA binding polyamides on oxime resin. Bioorg Med Chem (2002) 10(8):2767–74. doi: 10.1016/S0968-0896(02)00133-5 12057666

[16] Michl J Park KC Swietach P. Evidence-based guidelines for controlling pH in mammalian live-cell culture systems. Commun Biol (2019) 2:144. doi: 10.1038/s42003-019-0393-7 31044169 PMC6486606

[17] Frost P Moatamed F Hoang B Shi Y Gera J Yan H. *In vivo* antitumor effects of the mTOR inhibitor CCI-779 against human multiple myeloma cells in a xenograft model. Blood (2004) 104(13):4181–7. doi: 10.1182/blood-2004-03-1153 15304393

[18] Lehmann S Stiehl DP Honer M Dominietto M Keist R Kotevic I. Longitudinal and multimodal in *vivo* imaging of tumor hypoxia and its downstream molecular events. Proc Natl Acad Sci USA (2009) 106(33):14004–9. doi: 10.1073/pnas.0901194106 PMC2729010 19666490

[19] Koh WJ Rasey JS Evans ML Grierson JR Lewellen TK Graham MM. Imaging of hypoxia in human tumors with [F-18]fluoromisonidazole. Int J Radiat Oncol Biol Phys (1992) 22(1):199–212. doi: 10.1016/0360-3016(92)91001-4 1727119

[20] Gastelum G Kraut JA Poteshkina A Artiga E Weckstein G Frost P. Targeting of the hypoxia-induced acid microenvironment of multiple myeloma cells increases hypoxia-mediated apoptosis 59th annual meeting and exposition. Blood (2017) 130(Supplement 1):4376.

[21] Berra E Benizri E Ginouves A Volmat V Roux D Pouyssegur J. HIF prolyl-hydroxylase 2 is the key oxygen sensor setting low steady-state levels of HIF-1alpha in normoxia. EMBO J (2003) 22(16):4082–90. doi: 10.1093/emboj/cdg392 PMC175782 12912907

[22] Gastelum G Veena M Lyons K Lamb C Jacobs N Yamada A. Can targeting hypoxia-mediated acidification of the bone marrow microenvironment kill myeloma tumor cells? Front Oncol (2021) 11:703878. doi: 10.3389/fonc.2021.703878 34350119 PMC8327776

[23] Ortiz-Prado E Dunn JF Vasconez J Castillo D Viscor G. Partial pressure of oxygen in the human body: a general review. Am J Blood Res (2019) 9(1):1–14. 30899601 PMC6420699

[24] Warburg O. On respiratory impairment in cancer cells. Science (1956) 124(3215):269–70. doi: 10.1126/science.124.3215.269 13351639

[25] Giatromanolaki A Bai M Margaritis D Bourantas KL Koukourakis MI Sivridis E. Hypoxia and activated VEGF/receptor pathway in multiple myeloma. Anticancer Res (2010) 30(7):2831–6. 20683019

[26] Benej M Pastorekova S Pastorek J. Carbonic anhydrase IX: regulation and role in cancer. Subcell Biochem (2014) 75:199–219. doi: 10.1007/978-94-007-7359-2_11 24146381

[27] Lakota J Skultety L Dubrovcakova M Altaner C. Presence of serum carbonic anhydrase autoantibodies in patients relapsed after autologous stem cell transplantation indicates an improved prognosis. Neoplasma (2008) 55(6):488–92. 18999876

[28] Singh S Lomelino CL Mboge MY Frost SC McKenna R. Cancer drug development of carbonic anhydrase inhibitors beyond the active site. Molecules (Basel Switzerland) (2018) 23(5):1045. doi: 10.3390/molecules23051045 29710858 PMC6099549

[29] Supuran CT. Carbonic anhydrase inhibitors as emerging agents for the treatment and imaging of hypoxic tumors. Expert Opin Investig Drugs (2018) 27(12):963–70. doi: 10.1080/13543784.2018.1548608 30426805

