# Supplementary figures and images for: Acidification of intracellular pH in MM tumor cells overcomes resistance to hypoxia-mediated apoptosis in vitro and in vivo
Source: Front Oncol. 2023 Nov 3;13:1268421. doi: 10.3389/fonc.2023.1268421 (PMC10655143; doi:10.3389/fonc.2023.1268421)

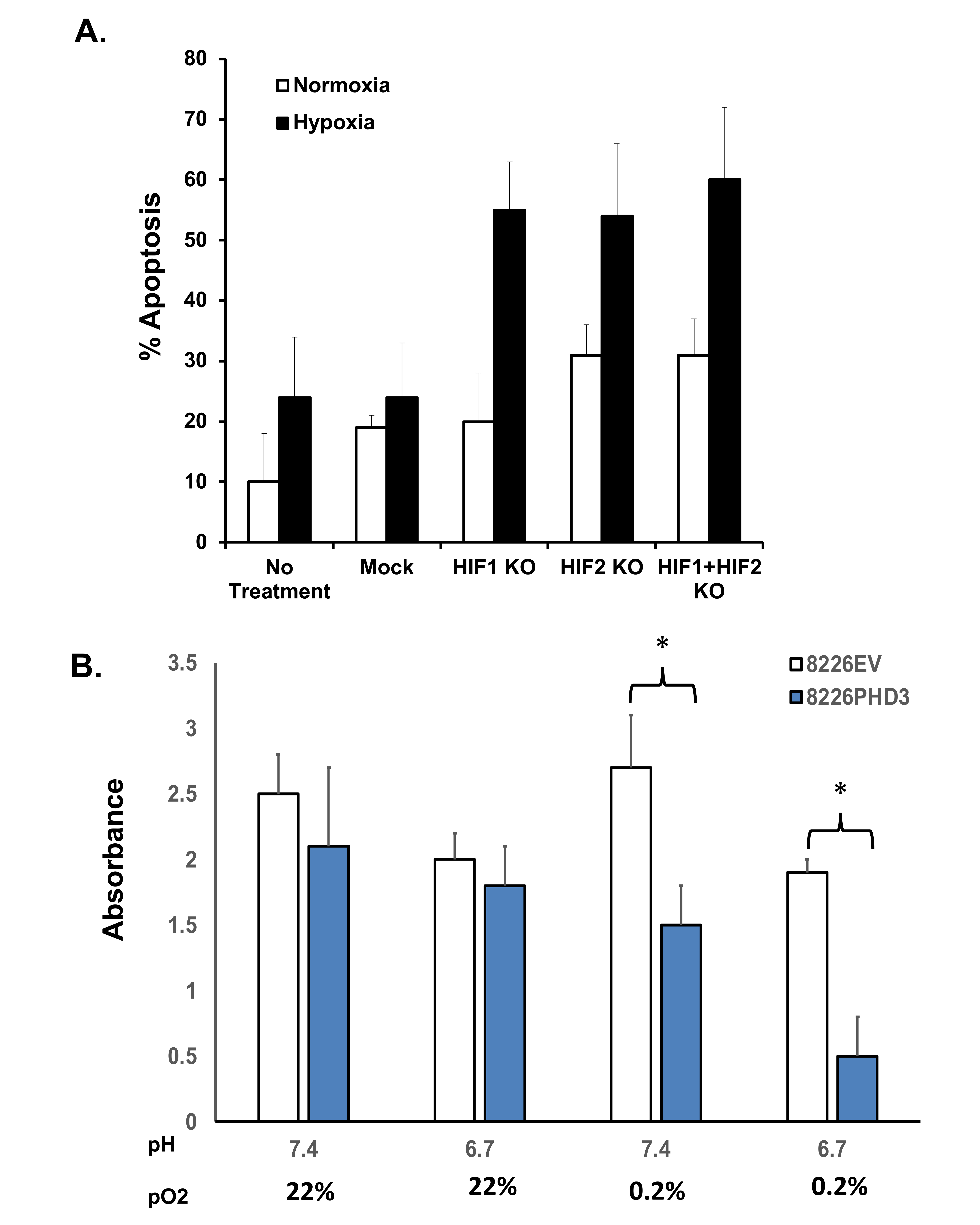

Supplement: Supplementary Figure 1 — Knockdown of HIFα-subunits sensitizes 8226 cells to hypoxia-mediated apoptosis. (A) 8226 cells were transfected with HIF1α siRNA, HIF2α siRNA or both HIF1α and HIF2α siRNA. Non-specific scrambled siRNA was used as a control. The cells were then cultured under normoxic (22% O2 white bars) or hypoxic (0.1% 02 black bars) conditions for 48 hours and apoptosis was measured by flow cytometry for cleaved caspase-3. The data shows mean ± SEM of 3 independent experiments. (B) Hypoxia reduces the growth of 8226PHD3 compared to 8226EV cells and this effect is augmented by acidic pH. WTS-1 assay was used to measure cell growth. Data is presented as mean ± 1 STD. * = p<0.05 (t-Test) for pairwise comparison of 8226EV (clear bars) compared to 8226 (blue bars). [file Image_1.jpg]

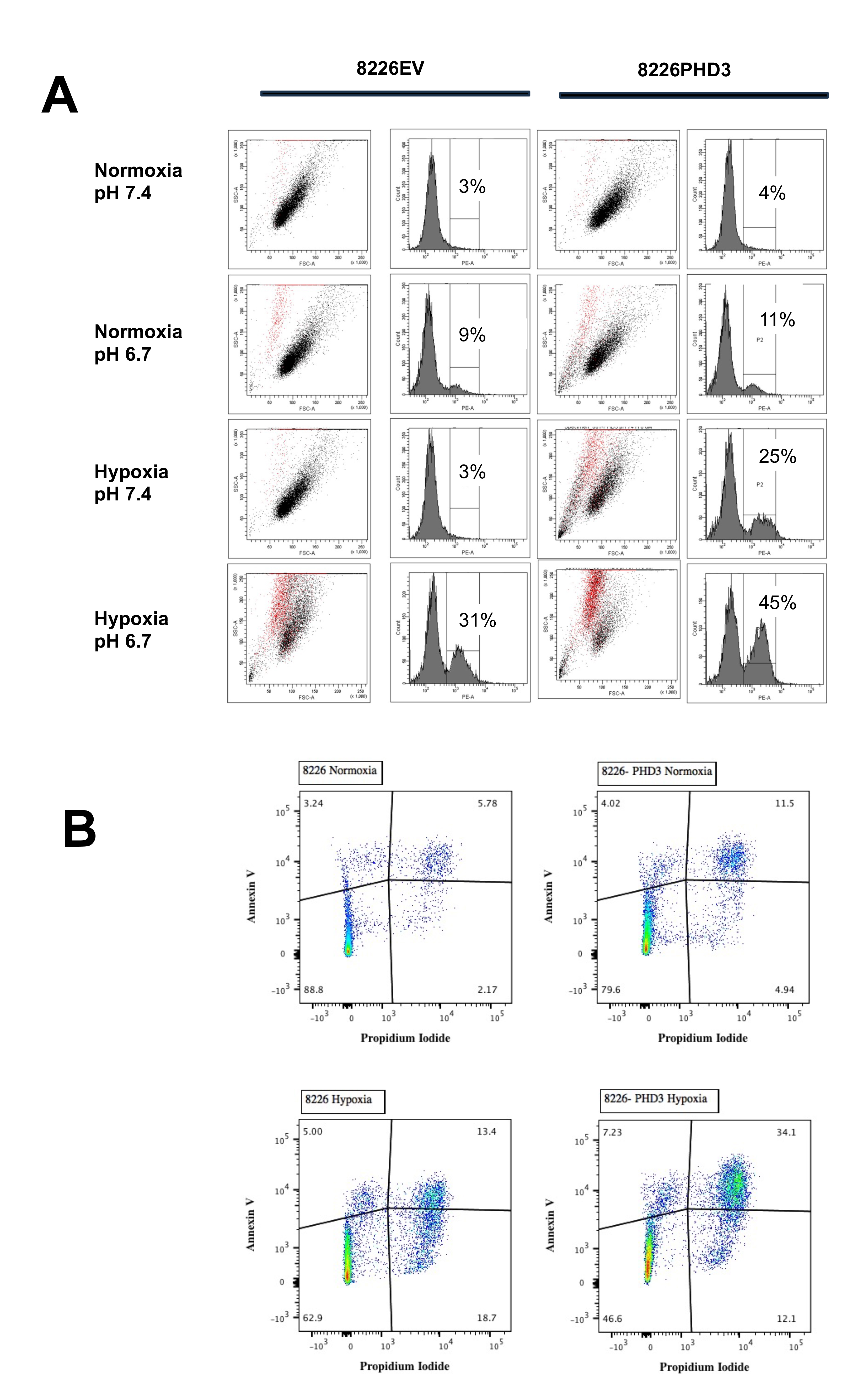

Supplement: Supplementary Figure 2 — Exogenous expression of PHD3 increases sensitivity of 8226 cells to hypoxia-mediated apoptosis under acidic conditions. (A) Representative histogram of isogenic 8226 cells cultured under variable pO2 and pH conditions 48 hrs. Apoptosis was measured by flow cytometry for cleaved caspase-3. The first set of panels show the forward scatter (FSC) plotted against the side scatter (SSC). The second set of panels shows the histogram of number of cells against the expression of PE and the gate used to differentiate between PE positive and PE negative cell populations. The values represent % apoptosis (PE positive) cells compared to total number of cells. (B) Apoptosis was measured in the same cells cultured as describe above, but with an Annexin V/PI apoptosis staining kit. [file Image_2.jpg]
